# The coevolution between telson morphology and venom glands in scorpions (Arachnida)

**DOI:** 10.1590/1678-9199-JVATITD-2020-0128

**Published:** 2020-10-09

**Authors:** Wilson R. Lourenço

**Affiliations:** 1 Muséum national d’Histoire naturelle, Sorbonne Universités, Institut de Systématique, Evolution, Biodiversité (ISYEB), UMR7205-CNRS, MNHN, UPMC, EPHE, Paris, France.

**Keywords:** Scorpion, Telson morphology, Venom glands, Coevolution

## Abstract

As in previous contributions to the *JVATiTD*, the aim of this note is to bring some general information on a particular aspect of the scorpion biology. An attempt is made to explain the possible coevolution of telson morphology and venom glands, which took place during several hundred million years and in particular since scorpions migrated from aquatic to terrestrial environments. Three components can be directly associated with predation and defensive behaviours: (1) morphology of the chelae and structure of the chelae fingers granulations; (2) morphology of the metasoma and in particular of the telson; (3) evolution of tegumentary glands in the telson toward different types of venom glands. Since a number of recent contributions already treated some of these aspects, I will limit my comments to the possible evolution of the telson in relation to the evolution of venom glands. As in previous contributions, the content of this article is basically addressed to non-specialists on scorpions whose research embraces scorpions in several fields such as venom toxins and public health.

## Introduction

**Figure 1.**
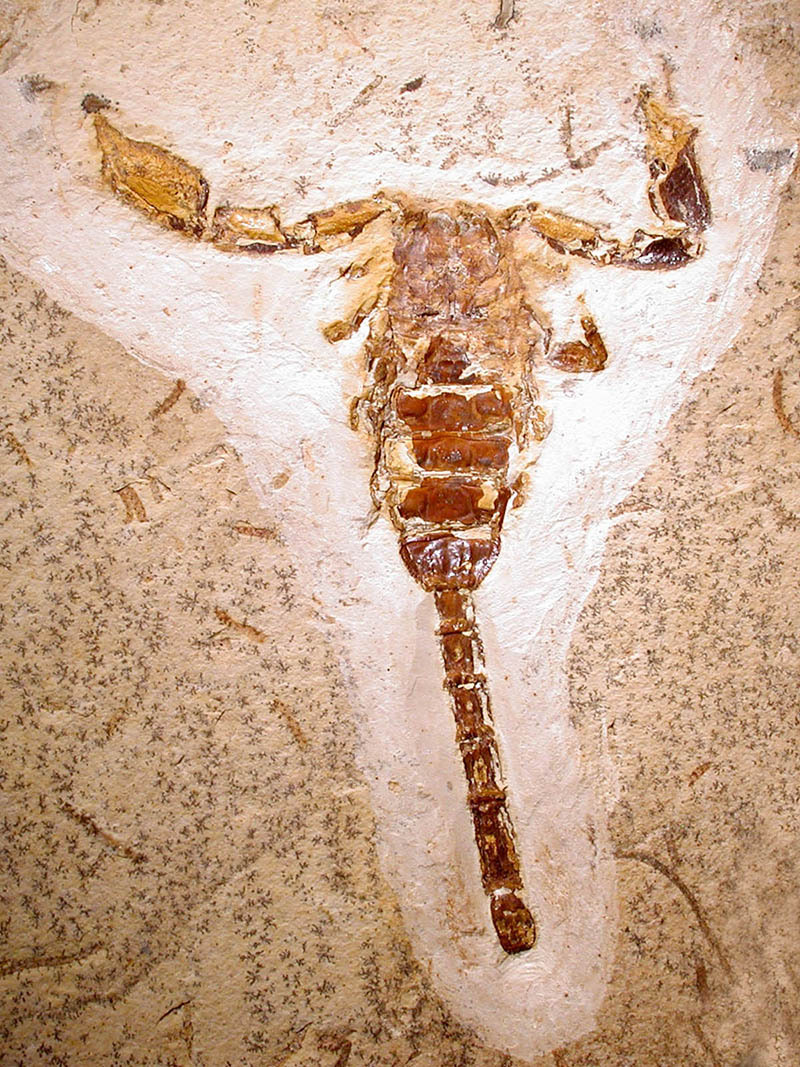
*Protoischnurus axelrodorum* Carvalho & Lourenço, early Cretaceous fossil from Araripe Basin in Brazil. Telson is bulbous without a sub-aculear tubercle.

It is well accepted by most authors that scorpions are among the most ancient and conservative arthropods both in origin and body morphology [1,2,3]. They first appeared as aquatic organisms during the Silurian (approximately 450 MYA) and apparently experienced few morphological changes since that period [1,2,3,4]. Recent discoveries even suggest that terrestrial forms probably occurred since the Silurian [5]. As consequence of their very conservative form (Figure 1), several authors suggested to define scorpions as ‘living fossils’. This definition, however, is not precise since scorpions certainly underwent major biochemical, physiological, behavioural and ecological adaptations that have combined to ensure their continued success over the past 450 million years and in particular their adaptation to land environments [1].

**Figure 2.**
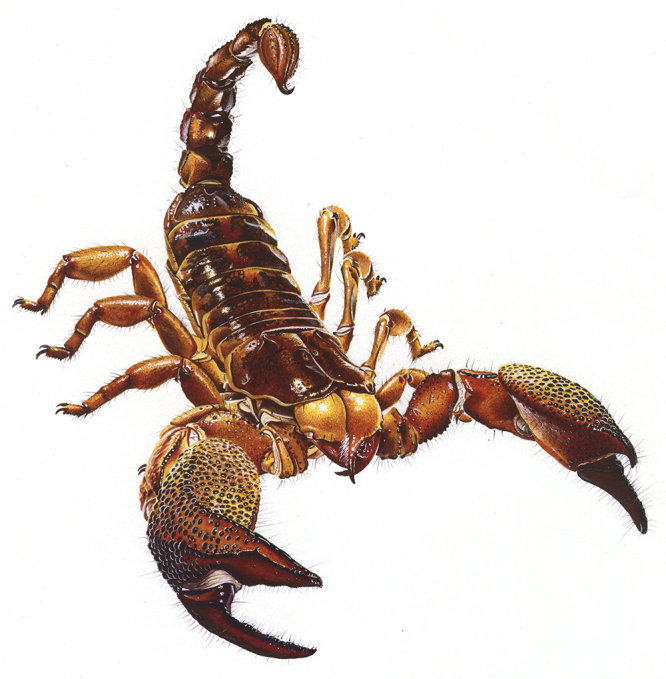
General anatomy of a scorpion. Habitus of *Pandinus imperator* (C. L. Koch), male from Africa (copyright by B. Duhem, reproduced with permission).

**Figure 3.**
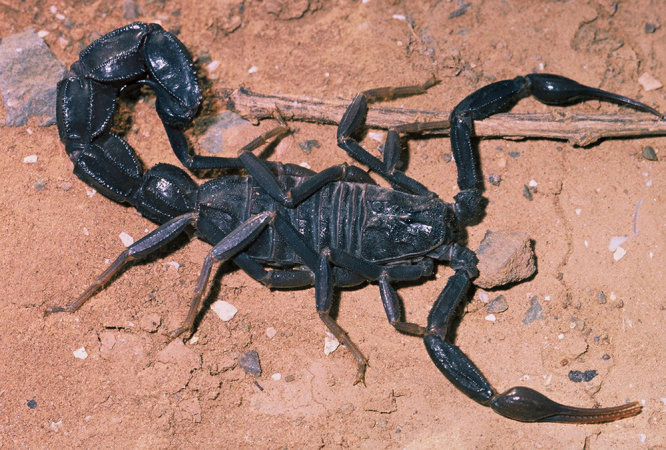
*Androctonus liouvillei* (Pallary), male from Maroc. A Buthidae scorpion with slender pedipalps and strong metasoma (copyright by M. Aymerich, reproduced with permission).

**Figure 4.**
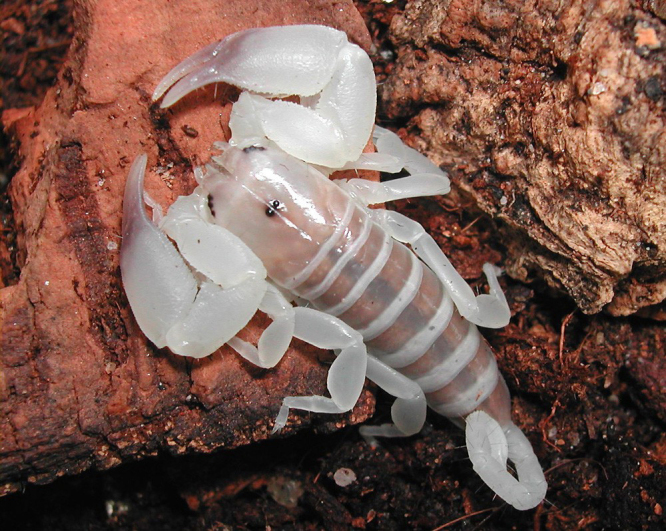
*Palaeocheloctonus septentrionalis* Lourenço & Wilmé, pre-adult male from Madagascar. A Hormuridae scorpion with bulky pedipalps and a diminutive metasoma (copyright by E. Ythier, reproduced with permission).

Although conservative, the general morphology of scorpions seems to be extremely well adapted to both predation and defensive behaviours. The body is almost totally articulated and composed of a fixed prosoma and an opisthosoma divided into 12 segments; seven that compose the mesosoma and five the metasoma (Figure 2). The morphology of metasoma can vary greatly among species of distinct families. In some species of Buthidae, metasomas can be very strong and bulky while in other families, such as the Hormuridae, metasomas can be extremely diminutive (Figures 3 and 4). In the extremity of the metasoma a telson is present, containing two glands. As for appendages, a pair of chelicerae, a pair of pedipalps and four pairs of walking legs are present. The general morphology of chelicerae varies mainly in the structure and number of teeth, but the morphology of pedipalps can vary greatly. These can be very short or extremely long; some are elongate slender others sturdy (Figures 3 and 4). Fixed and movable fingers of the pedipalps equally show marked differences in the granulations of their cutting edges. This structure is of major importance for the apprehension and capture of prey. The combination of all these morphological variations conduct to major differences in the general anatomy of scorpions which can also vary greatly in their global size which can range from about 1 to 25 cm. These variations in morphology often lead to more or less classical ‘clichés’ such as the one suggesting that scorpions with strong metasomas (fat tails) and weak pedipalps are globally dangerous while those with strong pedipalps and weak metasomas are harmless.

Scorpions are major predators but can also represent a selected prey for other predators [6,7]. Consequently, the evolution of predation and defensive strategies certainly represented a major aspect in the successful duration of their lineage [1,3]. Predation and defensive strategies depend mainly on three components:

- Morphology of the chelae and structure of the chelae finger granulations.
- Morphology of the metasoma and in particular of the telson.
- Evolution of tegumentary glands in the telson toward different types of venom glands.

Since some of these components have been correctly treated in a number of recent publications [8,9,10], the present note will focus mainly on the evolution of the telson morphology with possible correlations with venom glands [11]. It is recalled that as in previous publications for the *JVATiTD* the content of this article is basically addressed to non-specialists on scorpions whose research embraces scorpions in several fields such as venom toxins and public health. Consequently, its aim is not to treat the subject in an exhaustive way.

## The Evolution of Telson Morphology

The precise evolution of the telson remains unclear. The structure was already present in Eurypterids and is yet common in several arthropod groups such as Xiphosura (horseshoe crabs). This posterior-most division of the body of an arthropod is not however considered as a true segment since it does not arises in the embryo from teloblast areas as do real segments [3].

**Figure 5.**
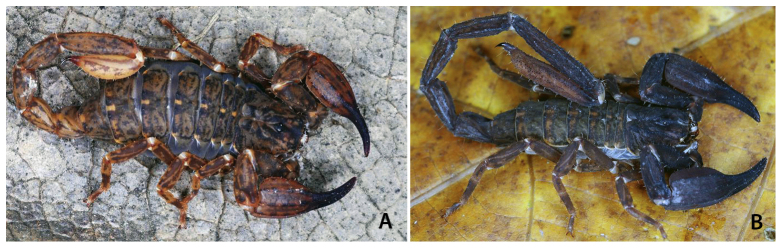
(A) Female and (B) male of *Chaerilus pictus* (Pocock) from India. A Chaerilidae scorpion showing a conspicuous sexual dimorphism of the telson (copyright by A. Zambre, reproduced with permission).

The morphology of most scorpions’ telson is rather similar, but some species may present huge particularities, both among species of the same genus or between sexes (Figure 5). Its basic morphology is composed of a vesicle that contains a pair of glands; this vesicle is prolonged by the aculeus, which bears two exit ducts, each corresponding to one of the glands. One aspect that calls the attention is the presence, in some groups, of a raised protuberance underneath the curvature of the aculeus. This protuberance may be a rounded tubercle, a sharp or rhomboid tooth or sometimes a more undefined structure [12,13].

This protuberance, generally called sub-aculear tubercle, most certainly evolved independently in two maybe three familial lineages [14]. This character was briefly discussed by a few authors, but very few comments were addressed on its possible function [9,13,14]. However, according to Van der Meijden and Kleinteich [9] the sub-aculear tubercle does not touch the surface of the prey/predator when the scorpion stings, consequently the possible function as an alternative pivot point to guide the initial penetration of the aculeus is rejected. They accept however, that very pronounced sub-aculear tubercles may contact the surface of the prey/predator after the tip has been sunk. For this scenario, the aculeus would have to be sunk into the surface of a prey/predator very deeply, situation not observed under laboratory conditions. Consequently, they suggested that the function of the sub-aculear tubercle remains unclear.

In the present note I tentatively suggest that the evolution of sub-aculear protuberances could be associated with the mechanical use of the telson and acted as a ‘brake’ to avoid damages to very long aculeus which could break during the penetration in the cuticle of potential prey/predator. Among several extant species, it is rather common to find scorpions with a broken telson in nature (Lourenço pers. obs. and Van der Meijden and Kleinteich [9]). To support this view, it seems important to propose a short review of the scorpion groups in which this particular morphological character is present, both in extant and fossil forms. A possible coevolution with glands producing more toxic venoms seems equally pertinent since among the most noxious known species, several present a regression of this morphological structure. This phenomenon was also observed during the ontogenetic evolution of several species [15]; aspect however globally ignored by subsequent authors.

**Figure 6.**
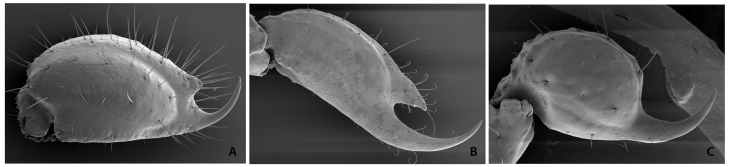
SEM images of telsons. **(A)** *Didymocentrus lesueurii* (Gervais) from Martinique Island, a Diplocentridae with a typical conic sub-aculear tubercle. **(B)** *Himalayotityobuthus martensi* Lourenço, Buthidae from Nepal with a huge sub-aculear tubercle. **(C)** *Kraepelinia palpator* (Birula), Buthidae from Iran with absence of a sub-aculear tubercle.

According to González-Santillán and Prendini [14], sub-aculear tubercles most certainly evolved independently in two, maybe three, familial lineages. Among extant groups of scorpions this structure is unequivocally present in all known species of the family Diplocentridae Karsch and in a large number of species of the family Buthidae C. L. Koch belonging to genera distributed in the Americas, Africa, Asia and Pacific islands (Figure 6). The sub-aculear tubercle seems further present and strongly developed in more basal lineages encompassing several genera such as *Ananteris* Thorell, *Ananteroides* Borelli, *Tityobuthus* Pocock, *Lychasioides* Vachon, *Himalayotityobuthus* Lourenço, *Lychas* C. L. Koch or *Isometrus* Ehrenberg. Within some less basal genera such as *Centruroides* Marx, *Rhopalurus* Thorell, *Microtityus* Kjellesvig-Waering and in particular *Tityus* C. L. Koch the degree of development of the sub-aculear tubercle can vary greatly ranging from very strong and robust to totally absent. The study of postembryonic developments of several species of *Centruroides*, *Tityus* and *Rhopalurus* equally revealed a regression of the sub-aculear tubercle during the ontogenetic evolution, which could even totally disappear in large adults [15]. Among the most evolved buthid genera such as *Androctonus* Ehrenberg, *Buthus* Leach, *Leiurus* Ehrenberg, *Buthacus* Birula or *Cicileus* Vachon, the sub-aculear tubercle seems globally absent.

The evolution of sub-aculear tubercles in other families such as the Vaejovidae and Chactidae may have a distinct origin and may be associated with smaller granules that form a compound sub-aculear tubercle [14]. In numerous species the ventral carina of telson can display strong granulations sometimes spiniforme. Some of these granules may be associated to the true sub-aculear tubercles.

**Figure 7.**
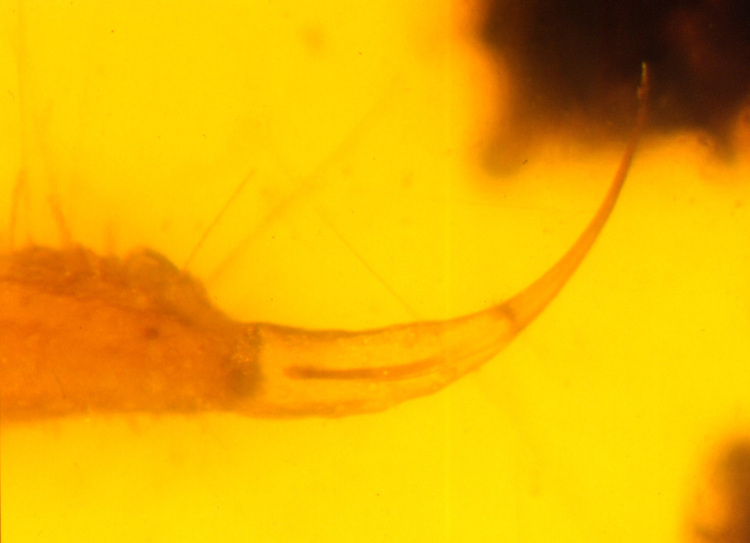
Slender telson with a long aculeus in the Cretaceous amber buthoid *Palaeoburmesebuthus grimaldii* Lourenço.

**Figure 8.**
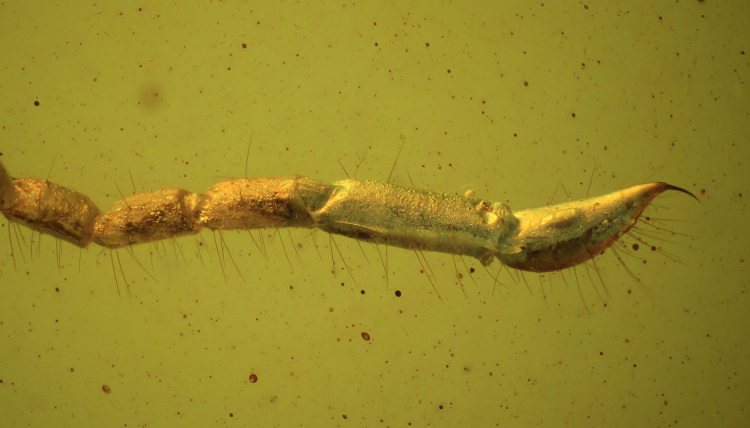
Bulbous telson with a short aculeus in the Cretaceous amber protoischnurid *Cretaceoushormiops knodeli* Lourenço.

**Figure 9.**
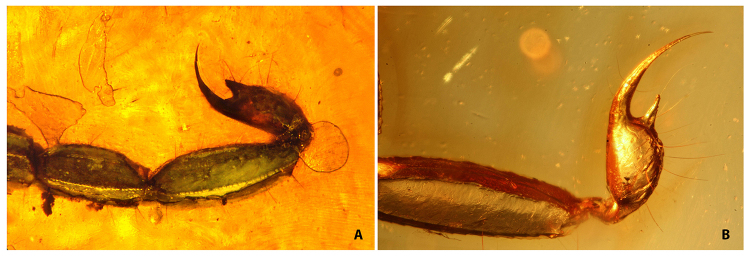
Telsons with strong developed sub-aculear tubercles on Cenozoic amber fossils. **(A)** *Palaeolychas balticus* Lourenço & Weitschat. **(B)** *Rhopalurus renelauerae* Lourenço.

Concerning fossils, for a majority of well-preserved specimens, the telson seems to lack any sub-aculear tubercle. For those from the Mesozoic to more recent periods, a similar pattern to that of extant forms is observed: Among buthoids slender telsons with very long aculei and among non-buthoids bulbous telsons with short aculei (Figures 7 and 8). One possible exception seems to be *Spinoburmesebuthus pohli* Lourenço, 2017 that shows one small sub-aculear tooth, but it could simply be a more developed granule of the ventral carina. Among Cenozoic fossils which, in most cases, are associated to extant lineages, very strong sub-aculear tubercles can be observed such as those of *Palaeolychas balticus* Lourenço & Weitschat and *Rhopalurus renelauerae* Lourenço (Figure 9).

## Conclusions

The original function of the telson in scorpions was most certainly mechanical playing a major role in predation. In this case, the aculeus acted as a ‘spear-head’. In most well studied fossil specimens from the Mesozoic up to more recent periods two major morphological types of telsons can be defined: (1) slender telsons with long aculei majorly associated with buthoid lineages, and (2) bulbous telsons with moderately long or short aculei majorly associated with non-buthoid lineages (Figures 7 and 8). Weak telsons with long aculei certainly were subjected to damages and could more easily broken during the penetration in the cuticle or skin of prey/predators. This situation probably positively selected the evolution of sub-aculear tubercles that most certainly evolved independently in different familial lineages [14]. The sub-aculear protuberances most certainly acted as a ‘brake’ to avoid damages to very long aculeus. During the Cenozoic period, the presence of sub-aculear tubercles seems to be predominant among buthoid lineages, which in most cases persisted up to present days. Among extant species, the representatives of these more basal lineages conserve the most conspicuous sub-aculear tubercles (e.g. genera *Ananteris*, *Tityobuthus*, *Lychas* or *Isometrus*), while more evolved groups knew a possible regression of sub-aculear tubercles until their total disappearance.

Non-buthoid lineages globally correspond to larger or at least more robust scorpions. Several species possess very strong pedipalps and predation can be performed just mechanically without the use of venom (Figures 2 and 4). In other words, they can capture prey without stinging. Besides, strong pedipalps are also used to intimidate small size predators. With the exception of the Diplocentridae family, the evolution of sub-aculear tubercles is almost absent from most non-buthoid lineages, probably because this morphological adaptation was not positively selected in absence of any particular selective pression. Consequently, the example of the Diplocentridae remains without a clear explanation and deserves further investigation.

**Figure 10.**
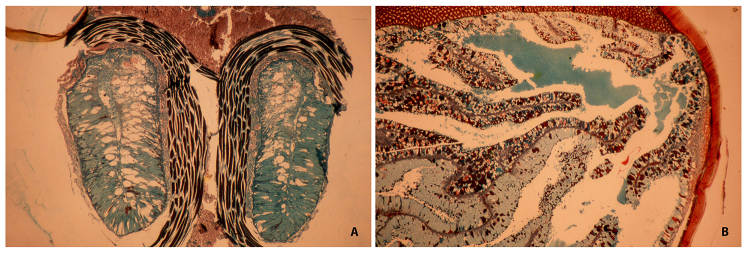
Histological sections of venom glands. **(A)** Simple, unfolded glands of *Opisthacanthus africanus* Simon, Hormuridae from Africa. Coloration used Trichrome de Gabe. **(B)** Complexly folded glands of *Leiurus hebraeus* (Birula), Buthidae from Israel. Coloration used Masson-Goldner.

All the extant scorpion species without exception possess venom glands. The presence of a telson with an aculeus and, in some cases, possibly tegumentary glands are also clearly evident in several fossil scorpions from the Palaeozoic, Mesozoic and Cenozoic [4,16,17,18,19,20,21]. Tegumentary glands are common in many arthropods and these probably evolved from the secretion of basic enzymes to more and more elaborate toxins, achieving to become complex venom glands (Figure 10). Based on the assumption that venom glands in scorpions have originally a predatory and digestive role, it is possible to suggest a process of coevolution between mechanical pattern of predation and the venomous function. The venom glands of Buthoid scorpions globally produce the most complex and elaborate toxins (Figure 10B). It is possible to suggest that these complex and more efficient toxins probably act as a driving selective pression on the morphology of extant telsons. This seems to be the case for several Old World genera comprising some of the most noxious species such as *Androctonus*, *Leiurus* and *Buthus*, which can be placed in a high or even very high evolutionary level within the buthoid lineage, but also that of some less noxious species belonging to the genera *Buthacus*, *Buthiscus* Birula and *Cicileus*. As for many Mesozoic fossil species these groups evolved again to telsons with quite long aculei [22,23]. The positive action of these powerful toxins most certainly authorized the evolution of possible more fragile aculei, which are, however, more performant to sting. In the case of these more evolved species, the sting can be performed very rapidly without less risks of damage [8,9]. One last factor probably contributed to the new evolution of long aculei in these groups; the presence of metals in cuticular structures. According to Schofield [24], heavy metals are found in several cuticular structures such as the sting (aculeus), mainly localized in regions susceptible to abrasion and mechanical force through contact with the environment. Among most studied organisms, scorpions showed the highest metal concentrations and the greatest variety of metal enrichment in their cuticle. Only extant species have being studied but it can be suggested that this cuticular reinforcement positively evolved since the Mesozoic and Cenozoic periods with the achievement of much more solid aculei.
